# Sandwich Face Layer Debonding Detection and Size Estimation by Machine-Learning-Based Evaluation of Electromechanical Impedance Measurements

**DOI:** 10.3390/s23062910

**Published:** 2023-03-07

**Authors:** Christoph Kralovec, Bernhard Lehner, Markus Kirchmayr, Martin Schagerl

**Affiliations:** 1 Institute of Structural Lightweight Design, Johannes Kepler University Linz, 4040 Linz, Austria; 2 Silicon Austria Labs GmbH, 4040 Linz, Austria

**Keywords:** sandwich debonding, detection and size estimation, electromechanical impedance method, machine learning, feature engineering, physics-based

## Abstract

The present research proposes a two-step physics- and machine-learning(ML)-based electromechanical impedance (EMI) measurement data evaluation approach for sandwich face layer debonding detection and size estimation in structural health monitoring (SHM) applications. As a case example, a circular aluminum sandwich panel with idealized face layer debonding was used. Both the sensor and debonding were located at the center of the sandwich. Synthetic EMI spectra were generated by a finite-element(FE)-based parameter study, and were used for feature engineering and ML model training and development. Calibration of the real-world EMI measurement data was shown to overcome the FE model simplifications, enabling their evaluation by the found synthetic data-based features and models. The data preprocessing and ML models were validated by unseen real-world EMI measurement data collected in a laboratory environment. The best detection and size estimation performances were found for a One-Class Support Vector Machine and a K-Nearest Neighbor model, respectively, which clearly showed reliable identification of relevant debonding sizes. Furthermore, the approach was shown to be robust against unknown artificial disturbances, and outperformed a previous method for debonding size estimation. The data and code used in this study are provided in their entirety, to enhance comprehensibility, and to encourage future research.

## 1. Introduction

Modern products face increasing demand for sustainable manufacturing and operation. These demands are addressed by research in a variety of technological fields, amongst which, lightweighting of the mechanical structure of a product is often applied. Applying high-performance materials, such as fiber-reinforced polymer composites, and modern numerical design methods, such as the finite element (FE) method, enables the highest optimization of mechanical structures. However, high optimization always implies the possibility of critical failure, e.g., when loads are underestimated, when unexpected manufacturing defects exist, or when damages occur during operation. These uncertainties are a significant issue in industries where the highest structural reliability is requested, e.g., transportation industries. To address these uncertainties, e.g., in aviation engineering, damage tolerance design philosophy is used together with maintenance programs: this produces high operational costs, due to downtime and labor. An upcoming approach to reducing maintenance costs, while reliably obtaining integrity, is structural health monitoring (SHM) [1,2].

SHM is the continuous on-board monitoring of the condition of a mechanical structure, during operation, by online and integrated systems of sensors, and can be classified into five levels: (i) damage detection; (ii) damage localization; (iii) damage quantification; (iv) damage typification; and (v) structural integrity assessment. Numerous active and passive SHM methods readily exist, achieving up to level four in laboratory conditions [3,4,5]. These apply systems of sensors to measure different physical effects, and continuously evaluate the collected data for potential damage. Among the various methods, acoustic methods—such as the passive acoustic emission (AE) method or the active guided waves (GW) and electromechanical impedance (EMI) method—are of the highest research interest, due to their sensitivity to structural change, and their potential for damage identification up to level four [2,3,5,6,7,8,9,10,11]. Furthermore, the often-used piezoelectric wafer active sensors (PWAS) are lightweight and easy to apply; however, due to the high sensitivity of acoustic methods, they are also very prone to environmental influences, measurement equipment, etc. This makes their application in realistic operational conditions extremely challenging, and has thus, to date, prevented their widespread industrial application. This is particularly true for the EMI method, which evaluates the dynamic response of a structure of interest (part of an electromechanical system) to continuous harmonic excitation, i.e., it is a vibration-based method. Consequently, the evaluation of potential damages is extremely complicated, due to reflections from more distant structural regions, e.g., boundaries and correlated disturbances [12]. Classical statistical damage metrics typically do not allow reliable damage identification [9]. However, recently, with the development and increasing distribution of novel statistical methods like machine learning (ML), and the availability of large computational power, new potential to solve the issue has been identified by the scientific community [13]. This has been similarly reported by Avci [14] for “vibration-based methods”, by a review of a number of selected journals. Furthermore, numerous recent review articles have highlighted the application and the advances of ML-based measurement data evaluation for vibration-based damage monitoring methods, such as the EMI [13,14,15,16]. Fan [13] classified EMI measurement data evaluation methods into data-based and physics-based methods.

Significant advances in data-based methods, which rely solely on real-world measurement data, are currently being achieved, in light of the increasing number of damage indicators, via the application of novel ML algorithms. Lopes [17] used an artificial neural network (ANN) to evaluate EMI measurements for monitoring the loosening of bolted joints of a scaled steel bridge section. The algorithm evaluated the data of four PWAS in two steps, and succeeded in detecting and locating loose joints, and identifying the severity of the loosening (one, two, or three bolts loose per joint). The training was done according to the same structure, by systematically loosening and tightening bolts. Giurgiutiu [11] demonstrated, in laboratory conditions, the superiority of a probabilistic neural network (PNN) to conventional damage-metric-based EMI measurement data evaluations, by means of artificially introduced cracks in an aging aircraft panel. He applied a number of the largest resonance frequencies as features, and classified unknown data into pristine and non-pristine (i.e., damaged). For training, only pristine state data was used. While the classical damage metric was only capable of detecting a crack in a very narrow region, the PPN-based evaluation also identified all cracks, at a distance of 100 mm. Another demonstration of PNN-based EMI measurement data evaluation for damage was reported by Palomino [18], who investigated a number of different damage scenarios in laboratory conditions, and was able to detect, localize, and classify cracks and rivet losses in large aluminum aircraft fuselage components by means of a sensor array consisting of eight PWAS. Park [19] demonstrated the potential of principal component analysis (PCA) to extract relevant features for damage monitoring from EMI measurement data: he used a bolted metal–metal single-lap shear joint, and used bolt loosening and bolt re-tightening as damage. He clearly showed the improvement of the feature extraction by the PCA, for differentiating between a loose and a re-tightened joint by a simple damage metric, and he also demonstrated the applicability of the k-means clustering algorithm to damage identification, using only two principal components. Min [20] addressed the sensitivity of EMI signal damage metrics to the considered frequency ranges, and solved the issue by training an ANN with damage metric values of discretized frequency spectra, to identify five different damage scenarios at a building structure (zero to four loose bolts at a joint). The same approach also performed well in an experimental study on the detection of loose bolts and cracks induced on a steel bridge. Oliveira [21] first proposed a convolutional-neural-network(CNN)-based evaluation method for damage detection, by the use of EMI measurements, and demonstrated its superiority to other ML methods on an aluminum plate equipped with three PWAS and with nuts adhesively bonded to it as artificial damages. Rezende [22] used aluminum beams, with weight added as damage, at varying temperature conditions, and demonstrated CNN-based evaluation also for temperature compensation. A similar demonstration was performed by Li [23] for the more complex use case of a concrete cube with cracks at varying temperature conditions, using CNN in combination with Pearson Correlation Coefficient indices from measured electromechanical admittance spectra sub-ranges, to identify both crack length and applied temperature. Furthermore, Li [23] used the Orthogonal Matching Pursuit algorithm to synthetically generate data based on measurement data, to reduce training costs for the CNN. Other successful applications of CNN models were reported by Ai [24,25], who demonstrated the identification of concurrent compressive stresses and damage (crack number and width), and minor mass loss of a concrete specimen. The application of a deep residual network to the EMI data evaluation for damage, and its potential to overcome the necessity of programmer-dependent preprocessing, was demonstrated by Alazzawi [26], who managed to directly evaluate time domain impedance data for crack quantification and localization in a steel beam. This potential for the evaluation of electromechanical admittance raw data was also found for a deep neural network by Nguyen [27], who applied it to the EMI data-based prestress loss monitoring of a post-tensioned reinforced concrete girder, in laboratory conditions. Singh [28] successfully used unsupervised self-organizing maps (SOM) to fuse multi-sensor data for the identification of hole sizes in a large aluminum plate. Furthermore, he found that data reduction of measured EMI spectra by PCA is not only more efficient but also improves the quality of the data for the realized experiments. The latter was also supported by the findings of Lopes [17], who stated that feature engineering, i.e., the selection of meaningful features of the raw data, often (i) highlights dynamic characteristics of the evaluation objective, and (ii) strongly reduces the required amount of training data. For a summary of the key contributions to data-driven and ML-based EMI measurement data, see Fan [13].

Physics-based methods use physical models of the monitored structure of interest and the applied sensor system, to evaluate real-world EMI measurement data. Analytical or numerical models are used to, e.g., find sensitive frequency ranges or specific sensitive features and their responses to a considered damage and its location and properties [29,30]. Another physics-based approach is model updating, whereby predefined parameters of a model, which represent damage, temperature changes, etc., are optimized until the EMI measurement data is matched by the model’s simulation results. Albakri [31], e.g., used a length-varying spectral element mesh to efficiently model a beam with a single crack-like opening, the opening parameters of which were subsequently optimized to fit the EMI measurement data of the equivalent, but physical, beam with a single unknown opening, thereby enabling the identification of the opening’s location, width, and severity. However, due to the large parameter space for more complex mechanical structures, and their large number of possible damage modes, the computational effort for model updating is large, and fast data evaluation is challenging. Ezzat [32] addressed this issue by increasing the efficiency of the model updating evaluation method by the application of surrogate models and a multi-stage statistical calibration framework. The framework used a pre-screening step to reduce the parameter space, by identifying probable damage locations before the model updating and signal matching steps and, therefore, strongly reduced the overall computational effort for damage identification. Another approach to accelerating data evaluation is to pre-simulate impedance spectra for varying damage parameters of the structure of interest. The typically large database is computationally expensive; however, its calculation can be done prior to the operational life of the monitored structure. To reduce this large computational effort, the application of numerical surrogate models (e.g., ML models) can be used to lower the number of required simulations by interpolation. An approach was recently demonstrated for the accurate interpolation of simulated guided wave signals by Humer [33]. The available simulated signals (and their correlated and known damage parameters) enable very fast damage identification, by simply comparing their similarity to measured EMI spectra. Shuai [34] and Kralovec [9] demonstrated this approach by the identification of the crack location and severity in a simple metallic beam, and the debonding size in an aluminum sandwich panel, respectively. However, physics-based methods are still limited to a low-frequency range or simple mechanical structures (mostly beams are investigated), as computational accuracy and efficiency are still issues [13].

Nevertheless, ML methods are generally found to be superior to traditional evaluation methods, in which the vibration signal is fuzzy and noise-contaminated [14,21]. To date, ML algorithms applied to vibration-based monitoring methods, such as EMI, are predominantly supervised learning algorithms based on real-world measurement data. Avci [14] concludes that the latest ML data evaluation approaches to potential damage increasingly apply signal features other than modal parameters, as these are often very sensitive to environmental conditions, e.g., temperature. Furthermore, the curation of experimental data is typically very costly. Thus, approaches using synthetic data (e.g., simulated by an FE model) are utilized, to reduce the cost of data acquisition [35,36]. Similarly, Fan [13] highlighted the significant potential of physics-based (i.e., based on synthetic data from physical models) EMI data evaluation, and the need for efficient numerical modeling.

The present research contributes to the field of EMI data evaluation for structural damage identification, by proposing a novel two-step physics- and ML-based EMI data evaluation approach to sandwich debonding detection and size estimation. The proposed novel approach addresses the above-mentioned research shortcomings by (i) very fast and accurate damage identification by supervised ML models, (ii) the replacement of costly real-world measurement data for training, by synthetic FE model-based data, and (iii) the engineering of EMI signal features that are robust against simplifications of the FE model (used to generate training data), environmental disturbances and small variations of the debonding shape (in reality, debondings are not perfectly circular). As a case example, a comparatively large and complex mechanical structure—a circular sandwich with aluminum face layers and an aluminum honeycomb core—was used. A single PWAS located at the center of the sandwich directly above the considered idealized face layer debonding was used for the EMI measurements. In contrast to many other EMI studies, the FE model used to simulate the artificial EMI data was strongly simplified, and was not intended to perfectly reflect all features of the real structural response: this, on the one hand, enabled a very efficient simulation of the artificial EMI data, which may be very relevant for larger structural components or more complex damage modes, and on the other hand, it represented a test of the proposed method’s robustness against incomplete training data. The considered idealized face layer debonding was increased stepwise by milling and EMI measurements, which were evaluated by the proposed physics-based ML approach. The approach was tested for the consistency, accuracy, reliability, and robustness of its evaluation results, and was benchmarked.

## 2. Electromechanical Impedance Data Generation

In the present research, we used synthetic and real-world EMI spectra as data. The synthetic EMI spectra were generated by a multi-physics FE model. The real-world EMI spectra were generated by measurements from a PWAS attached to a physical test specimen. To fully understand the EMI data generation, the present section presents (i) the fundamentals of the EMI method, (ii) the electromechanical system of the used case example of a circular sandwich plate with idealized face layer debonding and adhesively attached PWAS, (iii) the FE model and the conducted parameter study, and the generated data, and (iv) the real-world measurement setup and procedure, and the collected data.

### 2.1. Electromechanical Impedance Method

The EMI method is a vibration-based SHM method, which evaluates the dynamic response of a mechanical structure of interest to harmonic excitation. A single PWAS is usually used for both excitation and dynamic response measurement. The PWAS is adhesively bonded to the mechanical structure, resulting in the evaluated electromechanical system. For evaluation, the PWAS—and, thus, the whole electromechanical system—is excited over a wide frequency range by a harmonic voltage signal U(f), and the system’s dynamic response is measured by the PWAS’s electrical impedance Z(f)=U(f)/I(f) or its reciprocal, the admittance, where I(f) is the electrical current and *f* the frequency. Consequently, changes in the mechanical structure are also reflected in the measured impedance signal, and allow conclusions on its health state. The underlying correlation is given for one-dimensional strain and neglected PWAS dynamics by [2]

(1) $$Z\left(f\right)=\frac{1}{j\;2\pi fC\;}{\left[1-\kappa_{31}^{2}\frac{k_{\mathrm{st}}\left(f\right)}{k_{\mathrm{st}}\left(f\right)+k_{p}}\right]}^{-1},$$

where *C* is the capacity, $k_{p}$ the static stiffness, and $k_{31}$ the non-dimensional coupling coefficient of the PWAS. The behavior of the structure enters the equation by the frequency-dependent dynamic stiffness $k_{\mathrm{st}}\left(f\right)$. The imaginary unit is denoted by j.

### 2.2. Test Specimen

**Figure 1:**
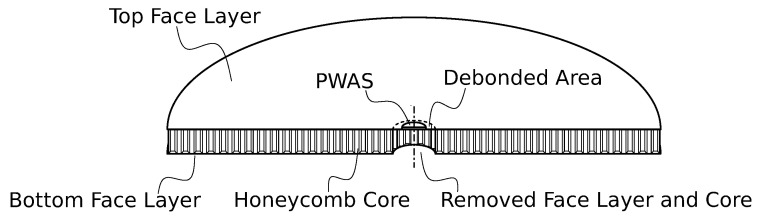
Circular sandwich panel with central face layer debonding and applied PWAS (cut and non-scaled visualization).

The investigated case example was the identification of sandwich debonding. A single circular aluminum honeycomb sandwich panel, with an idealized debonding in the center, was used in the present study. Figure 1 presents the used test specimen. The circular panel had the dimensions ⌀500×20 mm${}^{2}$. The face layers were produced from aluminum ENAW5754-H22, and had a thickness of 1 mm; thus, the aluminum honeycomb core (3/8–3000–0.0025) had a thickness of 18 mm. The in-plane orientation of the orthotropic honeycomb core was arbitrary, and was not considered in the present study. The face layer and core were adhesively bonded. To enable the physical introduction of debonding initiation and propagation, circular debonding was idealized by removing the core and bottom face layer in the debonded area. For the EMI measurements, a single PWAS of dimensions ⌀10×0.25 mm${}^{2}$ (PIC 255 material provided by PI Ceramic GmbH) was adhesively bonded (by LOCTITE EA 9466) to the center of the top face layer, i.e., directly above the debonded area. The material properties are presented later in Section 2.3.

### 2.3. Synthetic EMI Data Generation

The synthetic data were EMI spectra that were simulated by the variation of the material parameters of a number of multi-physics FE models that included different face layer debonding sizes.

#### 2.3.1. Simulation Model

**Figure 2:**
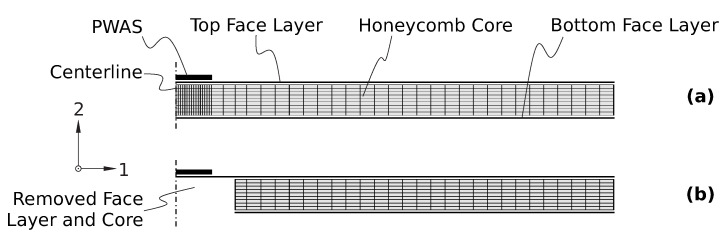
Axisymmetric FE model of sandwich panel in (**a**) pristine condition, and (**b**) with face layer debonding of a radius of 8.33 mm (non-scaled visualization).

The FE model was set up by the commercially available FE analysis software, Abaqus, provided by Dassault Systemes. The direct-solution steady-state dynamics analysis of Abaqus/Standard was used to simulate the dynamic response to harmonic excitation for the same 801 frequencies as measured on the physical test specimen (see Section 2.4). For efficiency, and to test the influence of unreflected features (e.g., all asymmetric modes and local resonances within core cells;, cf., [9]) in the training data, the FE model of the (in reality, orthotropic) sandwich structure was strongly simplified to an axisymmetric, and thus two-dimensional FE model. Consequently, the orthotropic honeycomb structure of the core was neglected, and was modeled by volume elements with homogenized transverse isotropic material properties $\left({\bar{G}}_{12}={\bar{G}}_{23}=\left(G_{12}+G_{23}\right)/2\right)$. Figure 2 presents the employed FE model.

The dimensions of the FE model were according to the considered test specimen (see Section 2.2). The face layers were modeled by quadratic, axisymmetric shell elements (Abaqus: SAX2). The core was modeled by quadratic, axisymmetric, and reduced integrated elements (Abaqus: CAX8R). The PWAS was modeled by quadratic, axisymmetric, piezoelectric, and reduced integrated elements (Abaqus: CAX8RE). The face layers were assumed to be perfectly bonded to the core and PWAS, and thus connected by tie constraints. The basic model discretization was based on a previous study that experimentally validated the model on an identical sandwich structure with idealized debonding damage. For further details of the FE model, see Kralovec [9]. The idealized debonding was modeled by simply removing the elements from the bottom face layer and the core, i.e., every damage size had its own FE model, where the remaining mesh was unchanged in respect of the pristine condition. Consequently, the simulated debonding sizes were modeled perfectly circularly, and were predefined by the model discretization. For the present work, the considered debonding sizes were given by their radius. Simulated debonding radii are $\left\{0,0.3125,\ldots \;5,6.6670,\ldots \;10,12,\ldots \;40\right\}$ mm: thus, 35 different FE models were used (including pristine condition). The material properties used for the FE models were typically from manufacturer data sheets; however, these always differed from the true properties, thus resulting in deviations between simulated and measured results. This could be addressed by, e.g., model updating during the initial pristine condition of a monitored structure of interest. However, due to, e.g., environmental conditions—such as temperature, moisture, or surface pollution—the properties of the structure could also vary during operation. To overcome these issues, we included possible variations of the material properties, via a large FE-simulation-based parameter study implemented by the software OptiSLang 8.0.0, provided by ANSYS Inc. Table 1 presents the considered material parameter ranges. The reference values were taken from manufacturer data sheets, or were typical values from the literature [2,9]. The parameter ranges represented extreme values found in data sheets and the literature, or were simply estimated within a spacious but meaningful range. The parameter range, in percent, was an additive scaling factor for all parameters in the table row, i.e., these parameters were assumed to change relative to one other, which (i) seemed reasonable, and (ii) strongly reduced the parameter space. The following are some arguments for the parameter range selection. The density of aluminum is exacted to be very constant, as it is defined by the alloy elements. However, the aluminum honeycomb core can be easily stretched or compressed during manufacturing: thus, its density and stiffness may vary together in a larger range. The Young’s modulus of aluminum typically varies in a narrow range. The review of different data sheets yielded the assumed parameter range. The Poisson’s ratio was found to be very constant for aluminum. Typical values for structural damping of mechanical structures are 0–0.05 [2]. The structural damping of aluminum is low. The aluminum honeycomb core was assumed to have stronger damping properties, due to the enclosed air. For the PWAS material parameter range, no adequate literature was found: thus, a small scattering was assumed to include the influence of these parameters.

To cover the material parameter space, space-filling Latin hypercube sampling was applied, with a sample size of 200: thus, a total of 35 damage states × 200 parameter variations = 7000 simulation results were generated (with 801 discrete dynamic frequency responses each). The overall computation time on a desktop machine was about 29 days. Using piezoelectric elements to model the PWAS in Abaqus, the dynamic frequency response was given by the frequency-dependent complex nodal charge output, $Q_{k}\left(f\right)$, where $k\in N_{e}$ were the nodes of the PWAS electrode supplied by the harmonic excitation voltage signal with constant amplitude $U_{\mathrm{fe}}$. The nodal charges could be used to calculate the complex impedance of the PWAS for the i=1…801 frequencies $f_{i}$, and thus, for the whole electromechanical system, by [9,29]

(2) $$Z\left(f_{i}\right)=U_{\mathrm{fe}}[j\;2\pi f_{i}(\underset{\mathrm{FE}\;\mathrm{result}}{\underset{︸}{\sum_{k=1}^{N_{e}}Q_{k}\left(f_{i}\right)}}\;-\underset{\begin{array}{c} \mathrm{electrical}\\ \mathrm{damping} \end{array}}{\underset{︸}{j\delta \epsilon_{33}^{T}\frac{U_{\mathrm{fe}}}{t_{p}}A_{p}}}){]}^{-1},$$

where $A_{p}$, $t_{p}$ and δ were the electrode area, the thickness, and the dielectric loss factor of the PWAS, respectively. The latter could also vary in a range of ±20%. Thus, varying the dielectric loss factor arbitrarily within this range enabled us to further multiply the synthetic data generated for training. In the present work, five arbitrarily selected values for the dielectric loss factor were used, thus yielding a theoretical number of 35,000 calculated EMI spectra. However, some of the generated parameter combinations did not give meaningful results, and were thus sifted out, yielding a final number of used synthetic EMI spectra of 34,152.

#### 2.3.2. Simulated Data

**Figure 3:**
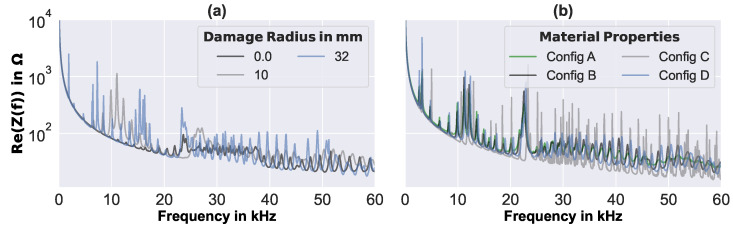
Examples of synthetic EMI spectra simulated by the FE model of the sandwich plate, with different arbitrarily selected material properties and (**a**) different debonding sizes, (**b**) debonding radius of 24 mm.

The FE-based simulation results were 34,152 complex EMI spectra of different perfectly circular debonding sizes and varying material parameters (cf., Section 2.3). Each of the simulated EMI spectra consisted of 801 complex values, which were evenly distributed over the measured frequency range of 0.004 kHz to 60 kHz (i.e., the simulated and measured results were at the same frequencies). For the selection of the material parameters, space-filling Latin hypercube sampling was used: thus, there was no sequence of simulation results in which the effect of a single parameter might be illustrated. However, Figure 3 shows the real part of a number of simulated EMI spectra, which reflect the influence of different debonding sizes (cf., Figure 3a) and the influence of the varied material properties for a constant debonding size (cf., Figure 3b).

For the circular plate with debonding, clearly higher and more resonance peaks can be observed than in the pristine case (i.e., damage radius 0; cf., Figure 3a). Furthermore, with debonding size increase, more resonance peaks appear at lower frequencies. Some of the first significant resonance peaks could be assigned to axisymmetric resonances of the debonded face layer, as demonstrated in [9]. Generally, it is difficult to conclude on specific effects of the debonding size on the simulated EMI spectra, as the material properties varied, and strongly influenced the baseline and resonance peak heights and frequencies (cf., Figure 3b). However, the intended representation of a wide range of material parameters in the simulated EMI spectra seems to have been achieved.

### 2.4. Real-World EMI Data Generation

The real-world data were EMI spectra that were measured on the considered test specimen with debonding sizes increased stepwise, and different artificial disturbances applied.

#### 2.4.1. Measurement Setup and Procedure

**Figure 4:**
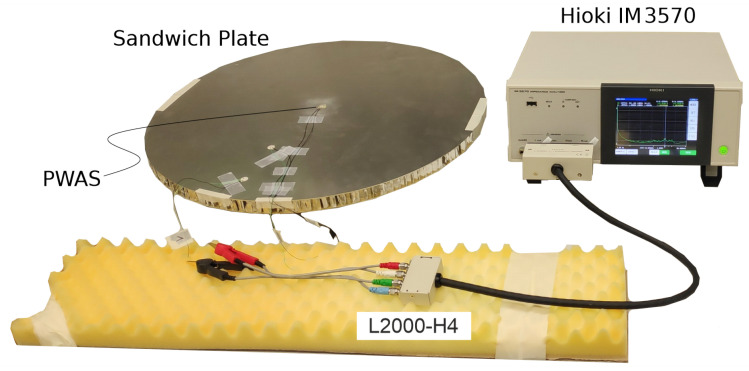
Setup for real-world EMI measurements.

The experimental setup for the real-world EMI measurements is presented in Figure 4.

**Figure 5:**
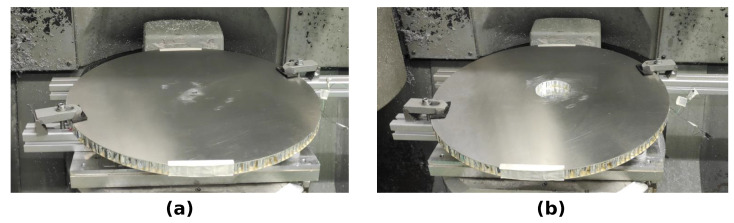
Circular sandwich plate clamped in the milling machine, with a debonding of the radius of (**a**) 2.5 mm, and (**b**) 40 mm.

The aluminum sandwich panel was placed on foamed polymer, to allow free vibration. The PWAS was electrically connected, via soldered 400 mm-long AWG 20 cables, to the four-terminal probe, type L2000, of the impedance analyzer, type IM3570—both provided by the Hioki E.E. Corporation. The excitation voltage amplitude of $5\sqrt{2}$ was constant over the whole considered frequency range of 0.004 kHz to 60 kHz. The frequency range was discretized by 800 evenly distributed steps, thus resulting in 801 complex impedance values per measurement. The excitation speed of the impedance analyzer was set to medium, and the averaging was set to three, to get a good balance between measurement noise and efficiency. In total, 225 EMI measurements were conducted, including both pristine and damaged states. The debonding was manufactured successively by removing, stepwise, the bottom face layer and the core, by milling. Figure 5 shows the clamped sandwich panel after tooling different debonding sizes in the milling machine.

The manufactured and measured 25 different debonding sizes are summarized in Table 2. The debonding shapes were mostly perfectly circular. Five debonding sizes had an elongated shape, which was manufactured by simply elongating the readily existing hole symmetrically along one arbitrary direction, until its largest extent had the value given in Table 2.

Furthermore, eight artificial environmental disturbances were used to influence the EMI measurements. The used disturbances were local forces and damping applied by human fingers, by a whole human hand, and by small modeling compound rolls. These disturbances were found, in previous experimental tests, to influence the measured EMI spectrum, and are summarized in Table 3.

**Figure 6:**
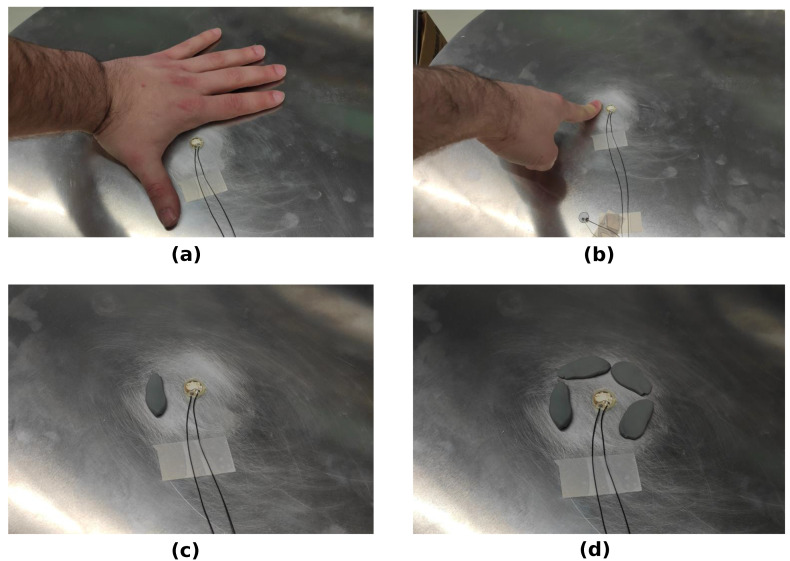
Examples of artificial disturbances: (**a**) hand next to PWAS; (**b**) one finger next to PWAS; (**c**) one modeling compound roll next to PWAS; (**d**) four modeling compound rolls next to PWAS.

Figure 6 shows four examples of the application of these disturbances.

For every debonding size, one EMI spectrum was measured without any disturbance, sequentially followed by EMI measurements with the disturbances presented in Table 3. Thus, nine measurements were done per debonding size, yielding a total number of 25 damage states × 9 disturbance states = 225 experimentally measured EMI spectra.

#### 2.4.2. Measured Data

**Figure 7:**
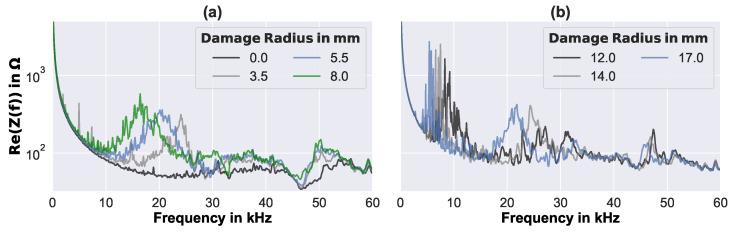
Examples of measured EMI spectra of the sandwich plate without any artificial disturbance, and with different perfectly circular debondings of (**a**) small size, and (**b**) middle-range size.

The results of the real-world EMI measurements were 225 complex impedance spectra of different debonding states and additional artificial environmental disturbances. Each of the EMI spectra consisted of 801 complex values, which were evenly distributed over the measured frequency range of 0.004 kHz to 60 kHz. Figure 7 presents the real part of a number of measured EMI spectra for (a) comparatively small, and (b) middle-range debonding sizes.

Readily small debonding sizes clearly changed the EMI spectra from the pristine state (debonding radius 0, cf., Figure 7a). With increasing debonding size, single very sharp resonance peaks appeared in the low-frequency range of the spectra (below 10 kHz). Furthermore, resonance frequency accumulations seem to have taken place around specific frequencies (e.g., at 17 kHz and 50 kHz for a debonding radius of 8 mm), yielding significant deviations from the pristine spectra. Moreover, the values of the presented EMI spectra generally increased with debonding size, and the resonances tended to lower in their frequencies. Similar trends may be seen also for middle-range debonding sizes; however, they are not clear anymore, as can be seen in Figure 7b. Similar experimental results were also found by the authors, in a previous work, for EMI measurements on identical circular sandwich plates with artificial debonding of a thickness of 20 mm and 40 mm, where the correlation between dominant resonance frequencies in the low-frequency range and the first eigenmodes of the debonded face layer was analytically, numerically, and experimentally demonstrated [9].

**Figure 8:**
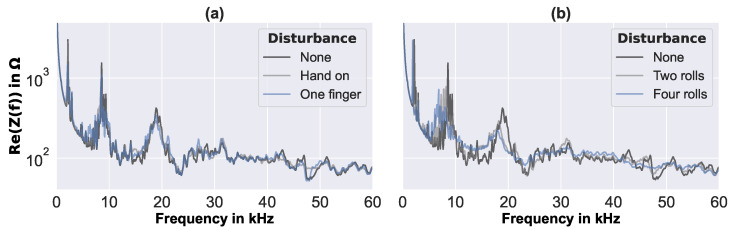
Examples of measured EMI spectra of the sandwich plate with perfectly circular debonding of the 29 mm radius, and (**a**) hand and fingers, (**b**) compound rolls applied as artificial disturbances.

In addition to the debonding size, other effects—such as measurement noise, ambient noise, pollution, and environmental temperature changes—may also have an influence on real-world EMI measurements [37,38]. Therefore, in the present work, strong artificial disturbances were applied, to test the robustness of the proposed evaluation method, and also to test some possible environmental influences during the operational life of a structural component. Figure 8 presents the real part of a number of EMI spectra measured on the circular sandwich plate, with perfectly circular debonding of the radius of 29 mm, and different applied artificial disturbances (cf., Section 2.4).

The effects of the artificial disturbances could be clearly observed in the measured EMI spectra. As expected, all applied disturbances resulted in a damping effect, that reduced the resonance peaks and smoothed out the spectra. For modeling compound rolls, the resonances additionally shifted towards lower frequencies, and the baseline of the EMI spectra appeared slightly increased. This may also have been due to the added mass. All the applied artificial disturbances clearly influenced the measured EMI spectra. Generally, the influence increased with the application of more fingers or more compound rolls. Furthermore, the influence of the artificial disturbances on the EMI spectra increased with the debonding size, in particular, when the disturbing fingers, compound rolls, etc., were readily located within the debonded area.

## 3. Data Preprocessing

A three-step approach was implemented for preprocessing the input data for the ML-based evaluation, in order to decrease its dimensionality and, at the same time, improve the robustness of the overall method: firstly, relevant frequencies were identified through analysis of the frequency spectrum, allowing for the elimination of unnecessary information; secondly, a log-scaled filterbank was applied to the data, leading to more robust representation, in terms of slight variations in resonance frequencies; lastly, a discrete cosine transform (DCT) was applied to the processed data, for further data compression and smoothing of the spectrum. After each step, it was important to confirm that enough relevant information was still present in the data. To ensure this, a well-known data exploration technique called t-SNE (t-distributed stochastic neighbor embedding) was applied. T-SNE is a non-linear, unsupervised technique for visualizing high-dimensional data in 2D or 3D maps, which preserves the local data structure: that is, data points close to each other in high-dimensional space will end up close to each other in low-dimensional space. This technique allows for the visualization of high-dimensional data in a lower-dimensional space, making it easier to identify patterns, and to confirm the preservation of relevant information. This way, the original data representation, with a dimensionality of 801, was reduced to only 31.

**Figure 9:**
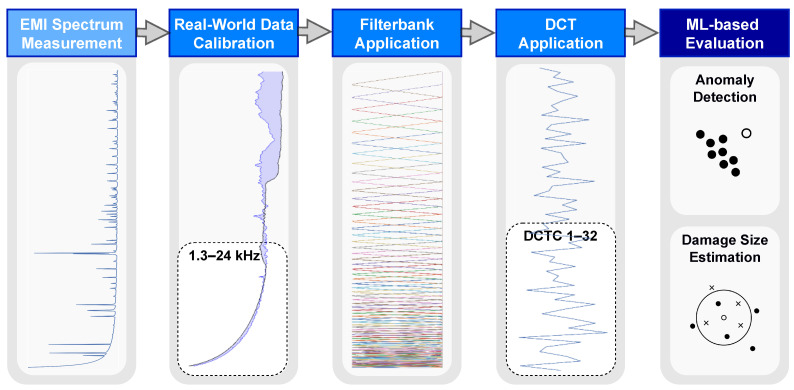
Flowchart of the preprocessing for real-world EMI data, where the original data representation, with 801 spectral bins (real part of impedance), was reduced step-by-step to only 31 DCT coefficients. The calibration process involved the adjustment of pristine real-world spectra to match pristine synthetic spectra. In the case that we only propagated a subset of the signal to the next step, it is outlined accordingly by a dashed window.

The features obtained through the preprocessing steps were then extracted from both the synthetic and the real-world EMI data, before conducting the ML-based damage evaluation experiments described in Section 4. The real-world EMI data were additionally calibrated to the synthetic data, to compensate for the strong simplifications of the FE model (see Section 2.3) used to generate the synthetic data. The finally defined preprocessing for the ML-based damage evaluation of real-world EMI data is depicted in Figure 9, for a better overview. The preprocessing for synthetic EMI data was the same, but without the calibration.

### 3.1. Feature Engineering

The goal of feature engineering is to improve the performance and robustness of ML-based algorithms: to this end, the dimensionality of data can be reduced for denoising purposes, and features can be developed to extract relevant information, while at the same time suppressing irrelevant information. The features used in this analysis were obtained through the process of feature engineering using exclusively synthetic data.

**Figure 10:**
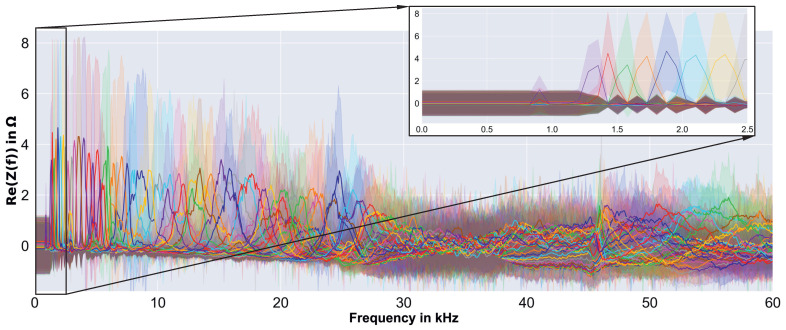
Summary of raw synthetic EMI spectra, scaled to have zero mean and unit standard deviation, for better clarity. The centerline is the mean value, and the surrounding translucent range represents the standard deviation per frequency of all spectra with the same damage size (approximately 1000 each).

We started with an exploration of the raw synthetic spectra, by looking at damage size-specific characteristics, to identify the most informative range of frequencies. In Figure 10, we can see the mean and standard deviation values of each damage size individually.

**Figure 11:**
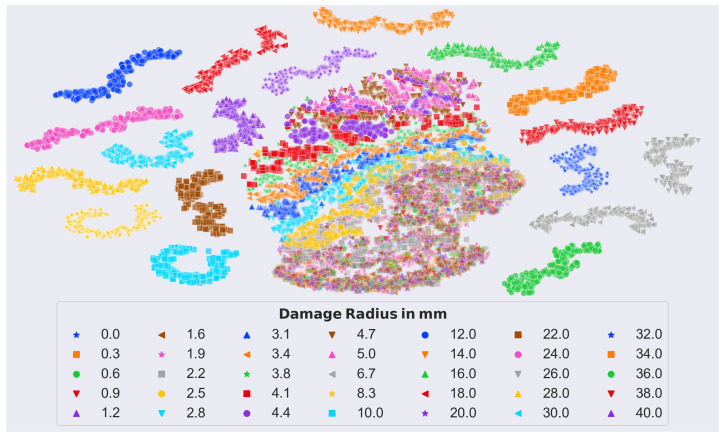
2D T-SNE embedding of synthetic data of the final frequency range from 1.3 kHz to 24 kHz, with a clear structure. The big cluster in the center comprises smaller defects up to 5 mm, and only very small defects (<2 mm) seem to overlap a lot (lower part of the center cluster).

Note that we scaled the spectra to have zero mean and unit standard deviation, for better clarity. In general, it can be seen that different damage sizes caused peaks (i.e., resonances) at different frequencies. However, frequencies below 1.3 kHz were found to be less informative (see Figure 10 detail), and were therefore excluded. Furthermore, the size-specific damage peaks were less pronounced, between 28 kHz and 46 kHz. Given our knowledge, from previous work, about the precision decrease of the simulation results with increasing frequency, and the clear objective to find an efficient evaluation method, we defined a frequency range between 1.3 kHz and 28 kHz to be considered, thereby readily shrinking the dimensionality of the data to less than half. With the help of the previously mentioned t-SNE plots, it was possible to further optimize the frequency range from 1.3 kHz to 24 kHz, i.e., only 38% of the available data. Figure 11 shows the clear structure of the t-SNE plot of the finally used frequency range.

The central cluster consisted of comparatively small damage sizes up to a radius of 5 mm. Furthermore, there was some overlap for very small damage sizes (debonding radius <2 mm) in the lower part.

After identifying the relevant frequencies, a filterbank was applied, which consisted of triangular filters that were 50% overlapping, and uniformly spaced on a logarithmic frequency scale. The overlapping of the filters allowed for a more fine-grained representation of the frequency spectrum, while the logarithmic spacing ensured that the filterbank was more sensitive to lower frequencies than to higher frequencies. This led to a more robust representation in terms of slight variations in resonance frequencies, while at the same time compressing the frequency axis of the spectrum. Additionally, log-scaling the magnitude compressed the dynamic range of the spectrum, which can be useful for handling signals with wide variations in amplitude. Considering the range of frequencies obtained in the previous step, and the filterbank configuration, a log-scaled spectrum with 66 bins was computed.

Finally, the log-scaled spectrum obtained through the previous steps was transformed, using the DTC [39]. The DCT is a mathematical technique used to decorrelate and transform data into a new coordinate system: that is, orthogonal cosine basis vectors with specific frequencies. The resulting transformed data will be a set of uncorrelated basis vectors, each representing a different frequency component of the original data. This step can further improve the robustness and efficiency of the data representation, by eliminating the dependencies between the data, furthermore only keeping the lower-order coefficients of the DCT results, in a signal that is more compact, and less affected by irrelevant information. This is based on the assumption that a signal can be separated—at least to a certain extent—into low-order components comprising relevant information, and high-order components that are irrelevant to the ML model, to perform the task at hand. In other words, the lower DCT coefficients represent a low-pass filtered spectrum that converges to its envelope. Furthermore, ignoring the 0th DCT coefficient increases the robustness of the method even further, by eliminating the dependency on the overall energy level of the signal.

The final feature vector consisted of 31 DCT coefficients that were computed on a log-scaled spectrum that exhibited a frequency range from 1.3 kHz to 24 kHz. This feature vector, which represented a robust and compact representation of the input signal, captured the essential spectral information relevant to damage identification. In the case of real-world data, there was an additional calibration step required, which is discussed in the next Section.

### 3.2. Calibration of Real-World EMI Data

**Figure 12:**
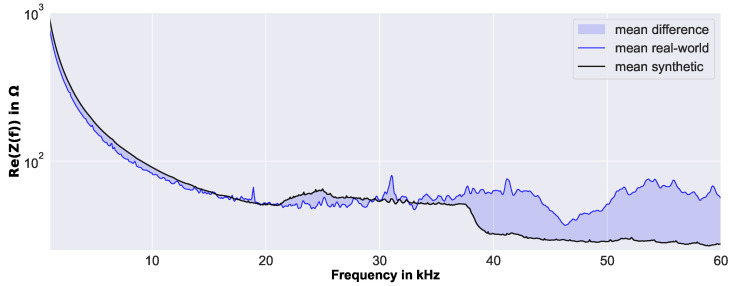
Difference between mean EMI spectra of all pristine real-world EMI spectra and mean EMI spectra of all pristine synthetic EMI spectra used to calibrate single real-world EMI spectra before evaluation.

The real-world data (i.e., the measured EMI spectra) were calibrated to the synthetic data (i.e., the simulated EMI spectra), to increase comparability. Therefore, it was possible to evaluate real-world EMI spectra by synthetic data-based features and ML models. This data preprocessing step was required, as the FE model used for the simulation of the synthetic EMI spectra was strongly simplified, to increase efficiency and robustness. The applied calibration was a simple shift of the real-world EMI spectra $Z_{i}^{r}$ by the difference of the frequency-dependent mean values of the real-world pristine data $\mu_{i}^{Z,r,p}$ and the synthetic pristine data $\mu_{i}^{Z,s,p}$, thereby aligning the pristine spectra of the real-world measurements and the synthetic simulations. For illustration, Figure 12 shows the mean real-world EMI spectra $\mu_{i}^{Z,r,p}$ for all measurements at the pristine sandwich panel, and the mean synthetic EMI spectra for all simulations by the pristine FE model $\mu_{i}^{Z,s,p}$.

Furthermore, the difference between the two mean spectra used to shift, and thus calibrate, the real-world data is highlighted. This difference was used to calculate the calibrated real-world EMI spectra by

(3) $$Z_{i}^{r,C}=Z_{i}^{r}-\left(\mu_{i}^{Z,r,p}-\mu_{i}^{Z,s,p}\right),$$

where *i* represents the bin of the spectrum (i.e., the 801 discrete frequency steps).

## 4. Data Evaluation Experiments

The data evaluation experiments tested a number of frequently used ML models for evaluating the existence and the size of a potential sandwich debonding. For a fair and meaningful comparison of the ML models, with respect to their performance, the available EMI spectra were split into arbitrary subsets, and the experiments were repeated at each of the subsets, thereby also enabling the evaluation of the consistency of the results, and thus the robustness of each ML model when repeated.

### 4.1. Debonding Detection by Anomaly Detection

In this Section, the results of the evaluation experiments for damage detection by anomaly detection methods are presented: firstly, the setup of the experiments is described; secondly, the results of synthetic EMI spectra are presented, i.e., the models are trained and tested by synthetic data; thirdly, the results of real-world EMI spectra are presented, i.e., the models are trained exclusively by synthetic data, but tested by calibrated real-world data.

#### 4.1.1. Setup of Experiments

The problem of anomaly detection was formulated as a novelty detection problem. The training data were not polluted by anomalies, and whether or not a new observation was an anomaly was evaluated. This could be done with the following models, which only needed normal data to learn their characteristics and identify deviations from this pattern as anomalies [40,41,42]:

1. Isolation Forest is an ensemble of binary trees specifically trained to isolate anomalies; decisions are made based on depth, where anomalous data points have shorter average depths in the tree structure;
2. One-Class Support Vector Machine (SVM) learns a boundary around the normal data points, which can be used to identify data points that deviate significantly from the normal data;
3. Elliptic Envelope assumes that the data are normally distributed, and fits an ellipse around them; the ellipse is determined by the mean and covariance of the data, and the assumption that anomalies are located in low-density regions.

For a fair and meaningful comparison, a cross-validation setting, which produced consistent results when experiments were repeated, was set up. The following setting was found to be adequate: the cross-validation used 128 splits, and 95% randomly picked EMI spectra from the pristine FE simulation model (synthetic data) as training data. For the experiments with synthetic data, the remaining 5% of the pristine, and all the anomalous EMI spectra examples, were used as test data. For the experiments with real-world data, only the real-world data were used as test data. All the models used the same cross-validation splits for training and testing.

#### 4.1.2. Results on Synthetic Data

**Figure 13:**
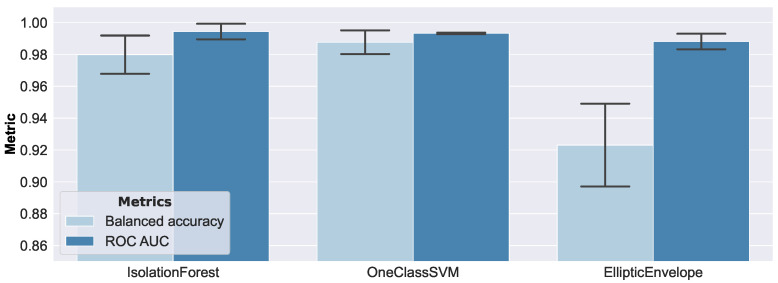
Anomaly detection results on synthetic data, by different ML models. According to balanced accuracy, the Isolation Forest and One-Class SVM seemed to perform reasonably well. The Elliptic Envelope exhibited the worst performance, due to a relatively high false positive rate.

Figure 13 presents the results of the anomaly detection methods on synthetic data.

**Figure 14:**
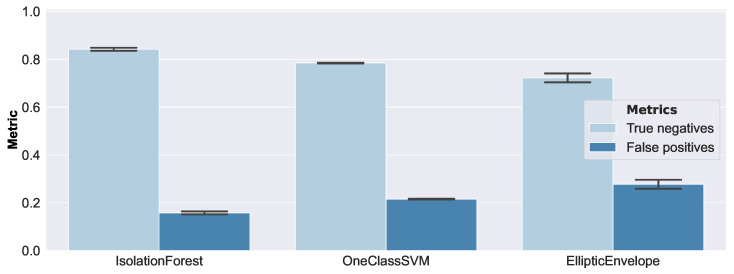
Anomaly detection results, on synthetic data, of small debonding radii of 2.2 mm and 2.5 mm. Larger damage was consistently detected accurately by all models.

The height of the bars refers to the mean value, and the error bars correspond to one standard deviation. According to balanced accuracy, the Isolation Forest and One-Class SVM seemed to perform reasonably well, while the Elliptic Envelope exhibited the worst performance. The ROC AUC metric did not reflect this as clearly, and should therefore be considered less appropriate in this setting. On closer inspection, the bad performance of the Elliptic Envelope was caused by a relatively high false positive rate on small damage sizes. A false positive example represented an anomalous example that was classified as normal (i.e., pristine), and a true negative example represented a correctly classified anomaly (i.e., damaged). Therefore, the false positive and true negative rates covered the performance of the complete set of anomalous examples, and always summed up to 1. In Figure 14, the false positive and true negative rates from the same experiment, with a focus on the comparatively small debonding radii of 2.2 mm and 2.5 mm, are shown.

In that regard, the Isolation Forest was the best-performing model, while the Elliptic Envelope had the poorest performance. Debonding sizes larger than a radius of 2.5 mm were accurately and consistently detected by all models.

#### 4.1.3. Results on Real-World Data

**Figure 15:**
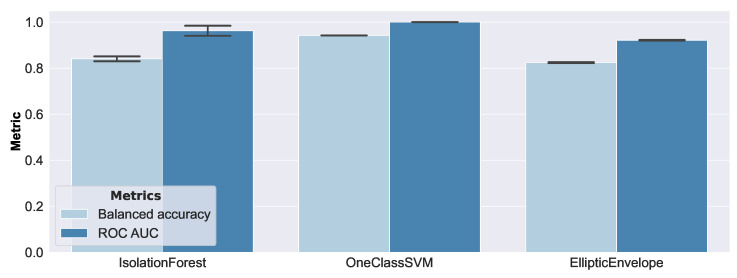
Anomaly detection results on real-world data.

Figure 15 presents the evaluation results of the real-world EMI spectra measurements.

**Figure 16:**
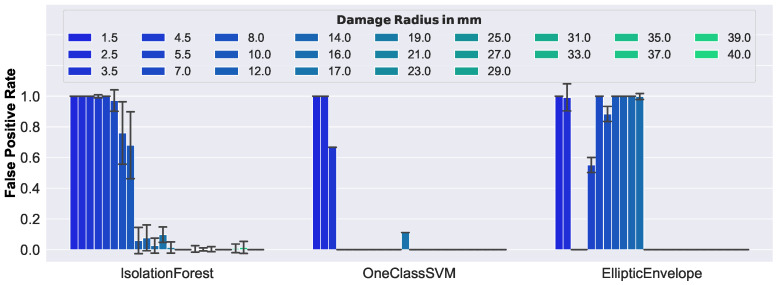
False positive rates of real-world data.

Interestingly, compared to the results of synthetic data, the ranking of the performance of the models was preserved, where the One-Class SVM was the best, and the Elliptic Envelope was the worst performing model. However, this time there was a greater difference in performance, with respect to the considered damage sizes. Figure 16 shows the false positive rate of each model, with respect to damage size.

As can be seen, smaller damages could not be correctly classified as anomalous. This was to be expected, given the geometric structure of the honeycomb core of the considered sandwich panel. The core material consisted of hexagonal cells with thin aluminum walls, whose core cells were oriented normally to the face layers, and had a size of 3/8″ (i.e., they enclosed a circle with a 4.75 mm radius). Furthermore, it can be confirmed that only the One-Class SVM consistently detected damage >3.5 mm, which was the reason for its best performance among the models. The Elliptic Envelope exhibited unexpectedly good performance on damage with 3.5 mm and 4.5 mm, but the reasons remain unclear at this time, and are still under investigation. According to these results, the One-Class SVM turned out to be the best-suited model, as the good performance of the synthetic data was well-aligned to the performance of the real-world data.

### 4.2. Debonding Radius Estimation by Regression

In this Section, the results of the evaluation experiments for debonding radius estimation by regression methods are presented: firstly, the setup of the experiments is described; secondly, the results of the synthetic EMI spectra are presented (i.e., the models trained and tested by synthetic data); thirdly, the results of real-world EMI spectra are presented (i.e., the models trained exclusively by synthetic data, but tested by calibrated real-world data), and are compared to a baseline previously published by Kralovec [9].

#### 4.2.1. Setup of Experiments

The regression problem, where the debonding size had to be estimated based on the EMI measurement data, was realized by a baseline algorithm (BASE; a simple nearest-neighbor-based approach proposed by Kralovec [9], which works directly on the spectra of the measurements) and the following frequently used algorithms [43,44,45]:

1. K-Nearest Neighbor (KNN), which selects the K-Nearest Neighbors, based on a chosen distance (we used Euclidean distance and K=1), and outputs the corresponding target value;
2. Support Vector Regression (SVR), which is the counterpart of the nonlinear SVM algorithm for regression problems;
3. Multilayer Perceptron (MLP), which is a fully connected, feed-forward multi-layer neural network. We implemented one with just a single hidden layer of 48 neurons (MLP_48), and another with two hidden layers of 64 and 32 neurons (MLP_64.32).

The nearest-neighbor-based method (KNN) is interesting, insofar as the only difference to the baseline (BASE) is the representation of the measurement data: that is, the baseline takes the raw spectra as input, and the novel method takes the features computed from the raw spectra (data preprocessing) as input. Therefore, we could evaluate the impact of our feature extraction pipeline in the most meaningful way.

As with the previous debonding detection task (see Section 4.1), a cross-validation setting that produced consistent results when repeated was needed, to compare the models fairly. This time, the cross-validation used 32 splits, and 90% randomly picked examples as training data. For the experiments with synthetic data, the remaining 10% of the examples were used as test data. For the experiments with real-world data, only the real-world data were used as test data. All the models used the same cross-validation splits for training and testing.

#### 4.2.2. Results on Synthetic Data

**Figure 17:**
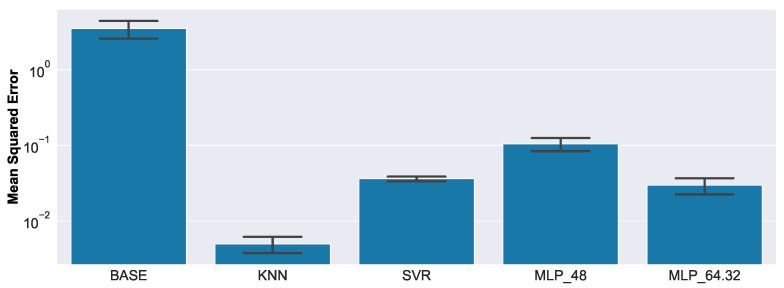
Regression results on synthetic data. Note the logarithmic scale on the *y*-axis. Compared to the baseline (BASE), all methods gave substantially better results, whereas the nearest-neighbor-based method (KNN) yielded the smallest overall MSE.

Figure 17 presents the regression results for the synthetic data set.

Compared to the baseline (BASE), all methods gave substantially better results, whereas the nearest-neighbor-based method (KNN) yielded the smallest overall Mean Squared Error (MSE). The MLP with higher capacity (MLP_64.32) predictably gave better results than the simpler MLP architecture with fewer neurons (MLP_48), and was on a par with the performance of the SVR.

**Figure 18:**
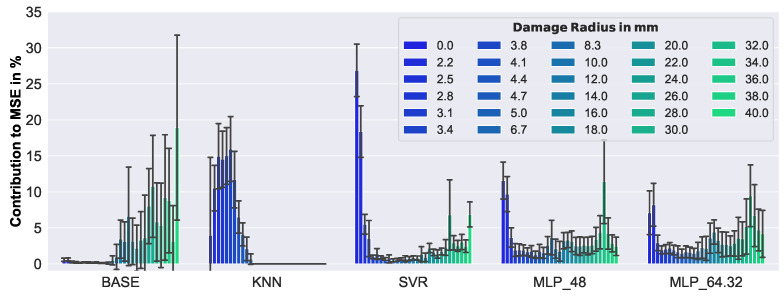
Relative contribution to the overall MSE, for each defect size, of synthetic data.

In Figure 18, a closer look at the results is given.

Here, the relative contribution to the MSE of each damage size is presented. Note that in such a visualization, the performance of the models cannot be compared in terms of MSE, but it sheds light on the behavior with respect to damage size. As can be seen, three different characteristics of the models can be distinguished: firstly, an increased error-proneness with increasing damage size (BASE); secondly, perfect handling of damage with ≥5 mm radius, and higher error-proneness with damage <5 mm radius (KNN); thirdly, higher error-proneness only with small (0–2.5 mm radius) and large damage (>30 mm radius) (SVR, MLP_48, MLP_64.32).

#### 4.2.3. Results on Real-World Data

**Figure 19:**
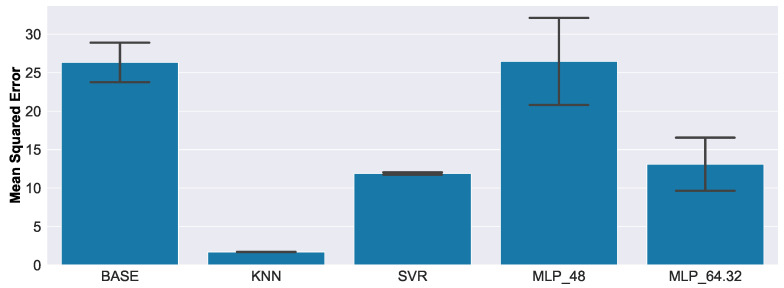
Regression results on real-world data. All methods except the baseline (BASE) and the smallest neural network (MLP_48) seemed to generalize reasonably well to unseen data.

Figure 19 presents the regression results of the real-world EMI measurements.

The MSE increased compared to the results on the synthetic data. This was expected, as no real-world data were used for training any of the models. All methods, except the baseline (BASE) and the smallest neural network (MLP_48), seemed to generalize reasonably well to unseen data. Similar to the anomaly detection task, the ranking between models, compared to the results of the synthetic data, was preserved, and the nearest-neighbor-based method (KNN) yielded the smallest MSE. Additionally, the results of KNN and SVR seemed to exhibit very small variances, which was also consistent with the results of the synthetic data. MLP_48 yielded the highest MSE, matching approximately the performance of the baseline (BASE).

**Figure 20:**
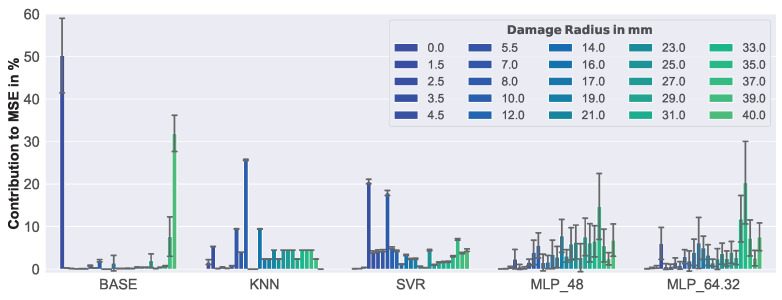
Relative contribution to the overall MSE, for each defect size, of real-world data.

In Figure 20, the relative contribution to the MSE of each damage size is presented.

Compared to the results of the synthetic data, the behavior of the models was quite different. To give an example, the contribution to MSE increased for the baseline (BASE) with defect size for synthetic data, but for real-world data, the highest contributions came from the normal examples with no defect, and the largest damage with 40.0 mm. On the other hand, the increased error-proneness with increasing damage size, which was noticed for the baseline (BASE) (see Figure 19), could now be observed in the MLP-based algorithms (MLP_48, MLP_64.32). The low variance that had already been observed with respect to MSE for KNN and SVR (see Figure 19) could also be observed here. This clearly indicates that the weaknesses of these models did not change, even when slightly different training data were used.

The erratic behavior of the baseline (BASE), especially for normal measurements—where often large damage sizes were predicted—rendered it unreliable. Given the overall low MSE and consistency of the results, the KNN algorithm seemed to be the best choice for damage size estimation, followed by the SVR. The MLP-based algorithms might perform better if more architectures were evaluated, but from those considered, only the more complex one (MLP_64.32) could outperform even the baseline.

## 5. Discussion

### 5.1. Evaluation Experiments

In the present research, great care was taken to meticulously select an appropriate cross-validation setup for each task individually, recognizing that this crucial step is overlooked in many studies. Thus, each experimental setup with cross-validation produced consistent results when repeated. Interestingly, the ranking of models was preserved between synthetic and real-world data for both tasks: this demonstrated that the synthetic data, in combination with the experimental setup, were sufficiently accurate for developing methods for a specific task, and for determining their relative performance in real-world settings.

### 5.2. Preprocessing

It is important to understand that although exclusively synthetic data were used to set up the complete preprocessing pipeline and ML models for evaluation, real-world data were still needed for calibration purposes. However, this was not a significant issue in practice, as the calibration process only required pristine baseline samples, which could be easily collected during the installation of the sensor network and the corresponding data acquisition system. Despite this, the value is recognized of further investigating more advanced preprocessing methods that could eliminate the need for calibration altogether.

### 5.3. Data

There are numerous disturbing influences on EMI measurements of operating mechanical structures, and thus challenges to the robustness of damage evaluation methods. The present research does not reflect all of these. Among them, measurement disturbances by ambient structural vibrations should be highlighted, as the results presented for the proposed two-step evaluation approach used comparatively low frequencies, and recent studies clearly show its relevance [38,46].

Thus, future research will address the approach’s robustness against ambient structural vibrations. Further frequently reported and not directly addressed influences on EMI measurements are temperature and moisture, as these are not expected to significantly affect aluminum sandwich panels. However, other important lightweight design materials, e.g., fiber-reinforced polymer composite, are well-known to be sensitive to temperature and moisture. The authors expect that an FE parameter study that also considers the density change of the face layers will reflect all parameters that are affected by temperature and moisture, and thus also allow damage evaluation in composite materials. This is also supported, as the real-world EMI data used for model testing already include measurements of unknown elongated debondings and artificial disturbances, which may have effects similar to temperature and moisture on composite properties. However, extended studies should also include composite materials and the examination of other non-idealized types of damages, such as delamination and cracks. The proposed physics-based two-step approach is expected to be applicable to all these cases, but may require inefficiently detailed FE models.

The considered debonding in the FE model is a strong idealization, and the results of the real-world measurements may also be interpreted as the identification of a hole in the back side of the sandwich panel. However, unpublished simulations with the same FE model, but with the remaining core and back-side face layer, show very similar characteristics of the EMI spectra. This is also supported by previous results of the authors [9], who found a clear correlation between the resonances in the EMI spectra and the debonded face layer area. Furthermore, damages distant from the sensor are expected to be very challenging to the proposed approach, due to the strongly increased parameter space. Nevertheless, today’s computational power, and the fusion of the data of a sensor array, are considered promising for solving this issue.

It is noteworthy that, while the real-world data used in this study provided valuable insights, the sample size was limited; therefore, the conclusions drawn from it should be considered within the scope of this limitation. Further research, with a larger and more diverse set of real-world data, is necessary, to fully validate the results and conclusions of this study.

### 5.4. Debonding Detection by Anomaly Detection

Depending on the material properties, small damages may only be detectable if a relatively high rate of false negatives (i.e., normal examples classified as anomalous) is accepted. However, by monitoring over a longer period of time, and evaluating summary statistics, satisfactory results may still be obtained. In regard to the real-world specimen used in this study, it might not be considered practical to investigate debonding damage sizes that are smaller than the size of the honeycomb core cells. However, the ability of the proposed method to identify even smaller damages could be useful in certain applications, such as predictive maintenance. These applications may also be supported by the short data evaluation times of the considered ML models, which are in the magnitude of $1/10^{-3}$ (for damage detection) and $1/10^{-5}$ (for damage size estimation) of the duration of a real-world EMI measurement.

Overall, the results are promising, and provide valuable insights into the use of anomaly detection models for identifying damage in sandwich panels.

### 5.5. Debonding Size Estimation by Regression

One of the main disadvantages of using the KNN approach is that it requires the traversal of the entire training data set during inference; therefore, the running time of a KNN is directly proportional to the number of examples in the data set, which means that the larger the data set, the more time-consuming the process will be. This can lead to increased computational time and memory usage, particularly when dealing with large data sets. In cases where real-time or near-real-time predictions are needed, the KNN approach may not be the most efficient choice, due to this requirement. If the computational efficiency is less important, the KNN results may be further improved by increasing K, the number of considered NNs. However, as most of the time the NNs correspond to the same target value, little improvement is expected. The ML models considered in the present research showed short data evaluation times in the magnitude of $1/10^{-3}$ of the duration of a real-world EMI measurement, and thus were hardly constrained in their possible applications. This efficiency may even question the need for the previous anomaly detection step. However, anomaly detection is (i) believed to be more robust in the evaluation of data that strongly deviate from the used training data, and (ii) 100-times faster, which appears meaningful for applications where normal data (data from the pristine structure) are predominantly evaluated.

## 6. Conclusions

The presented results clearly demonstrate the applicability of the proposed two-step physics- and ML-based EMI spectra evaluation approach for sandwich debonding detection and size estimation. The approach is capable of evaluating measured real-world EMI spectra exclusively based on synthetic, FE-simulated EMI spectra for debonding detection (step one) and debonding size estimation (step two). In addition, the feature engineering and the evaluation model development are conducted exclusively based on synthetic, FE-simulated EMI spectra. Therefore, the approach is very economic, compared to training with measured real-world data. The ML models applied for damage evaluation are, once trained, approximately $10^{5}$-times (for damage detection) and $10^{3}$-times (for damage size estimation) faster than the EMI measurement itself. This also enables quasi-real-time applications and edge computing at small and lightweight local processing units.

The best performance for damage detection by real-world EMI data was given by the combination of the developed data preprocessing with a One-Class SVM anomaly detection model. Misclassifications were only given for radial debonding sizes below 2.5 mm, i.e., debondings smaller than the honeycomb core’s cell size. The best performance for damage size estimation by real-world EMI data (1.7 mm MSE and 0.01 mm standard deviation) was given by the combination of the developed data preprocessing with a KNN regression model, which clearly outperformed a previous method used as a benchmark. Both the damage detection and the size estimation models were cross-validated, and showed high consistency in their results. Furthermore, the physics-based two-step approach demonstrated robustness against unknown artificial disturbances that were applied during real-world data acquisition. Data preprocessing was identified as the key factor in the performance of the proposed two-step evaluation approach, which—after comprehensive data exploration—found the consideration of a frequency range between 1.3 kHz and 24 kHz to be sufficient. This strongly reduced the ML model complexity, and allowed for a less discretized FE model, leading to a significant reduction in the simulation costs of the synthetic data.

However, the application of synthetic data-based features to the evaluation of real-world EMI measurement data still requires calibration to baseline measurements, to overcome the strong simplifications of the used FE model—a drawback that shall be addressed in future research. Future research must also investigate the proposed approach’s applicability to other important materials (e.g., composites) and their peculiar damage types, and damage evaluation with distant sensors. In addition, damage assessment with more remote sensors, and the extension of the approach to data fusion of a sensor network, need to be addressed.

The present work is also intended to promote future research in the field, by providing the complete EMI spectra data and the full code for data exploration, data preprocessing, and data evaluation (access see: Data Availability Statement).

## Figures and Tables

**Table 1:** Range for material parameter variations considered for the FE parameter study.

Parameter	Unit	Reference Value	Range	
face layer				
density	kg/m${}^{3}$	2660	–	
Young’s modulus	GPa	70	±3%	
Poisson’s ratio	1	0.33	–	
structural damping		0.01	0.0–0.05	
honeycomb				
density	kg/m${}^{3}$	59.3	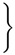	±18%
Young’s moduli $E_{11}$,$E_{22}$,$E_{33}$	GPa	0.634, 0.001, 0.001		
shear moduli $G_{12}$,$G_{23}$,$G_{23}$	GPa	0.137, 0.001, 0.275		
structural damping	1	0.02	0.0–0.05	
PWAS				
density	kg/m${}^{3}$	7800	–	
Poisson’s ratio	1	0.34	–	
elastic compliance coefficients $s_{11}^{E},\;s_{22}^{E}$	$m^{2}/N$	$\left\{16.1,\;20.7\right\}\times 10^{-12}$	±5%	
coupling factor for transverse oscillation	1	0.35	–	
relative permitivity $\varepsilon_{22}^{T},\;\varepsilon_{11}^{T}$	1	$\left\{1750,\;1650\right\}\;\varepsilon_{0}$	±5%	
piezoelectric large-signal deformation coefficient $d_{21},\;d_{22},\;d_{15}$	C/N	$\left\{-180,\;400,\;550\right\}\times 10^{-12}$	±5%	
dielectric loss factor	tan(δ)	0.02	–	

**Table 2:** Experimentally measured debonding sizes in mm.

0	1.5	2.5	3.5	4.5	5.5	7 *	8	10 *	12	14	16 *	17
19	21	23 *	25	27	29	31 *	33	35	37	39	40	

* elongated shape.

**Table 3:** Artificial environmental disturbances of EMI measurements.

Type	Approximate Total Load	Approximate Distance Plate Center to First Contact
whole hand	10 N	25 mm
one finger	10 N	25 mm
two finger	10 N	25 mm
three finger	10 N	25 mm
one roll	-	25 mm
two rolls	-	25 mm
three rolls	-	25 mm
four rolls	-	25 mm

## Data Availability

Data can be found at https://zenodo.org/record/6758723 (accessed on 23 December 2022). Code is available at https://github.com/berni-lehner/structural_health_monitoring/ (accessed on 23 December 2022).
